# Observed Measures of Negative Parenting Predict Brain Development during Adolescence

**DOI:** 10.1371/journal.pone.0147774

**Published:** 2016-01-29

**Authors:** Sarah Whittle, Nandita Vijayakumar, Meg Dennison, Orli Schwartz, Julian G. Simmons, Lisa Sheeber, Nicholas B. Allen

**Affiliations:** 1 Melbourne School of Psychological Sciences, The University of Melbourne, Melbourne, Australia; 2 Melbourne Neuropsychiatry Centre, Department of Psychiatry, The University of Melbourne and Melbourne Health, Melbourne, Australia; 3 Australian Centre of Posttraumatic Mental Health, Department of Psychiatry, The University of Melbourne and Melbourne Health, Melbourne, Australia; 4 Oregon Research Institute, Eugene, Oregon, United States of America; 5 Orygen, The National Centre of Excellence in Youth Mental Health, The University of Melbourne, Melbourne, Australia; 6 University of Oregon, Eugene, Oregon, United States of America; University of Missouri, UNITED STATES

## Abstract

Limited attention has been directed toward the influence of non-abusive parenting behaviour on brain structure in adolescents. It has been suggested that environmental influences during this period are likely to impact the way that the brain develops over time. The aim of this study was to investigate the association between aggressive and positive parenting behaviors on brain development from early to late adolescence, and in turn, psychological and academic functioning during late adolescence, using a multi-wave longitudinal design. Three hundred and sixty seven magnetic resonance imaging (MRI) scans were obtained over three time points from 166 adolescents (11–20 years). At the first time point, observed measures of maternal aggressive and positive behaviors were obtained. At the final time point, measures of psychological and academic functioning were obtained. Results indicated that a higher frequency of maternal aggressive behavior was associated with alterations in the development of right superior frontal and lateral parietal cortical thickness, and of nucleus accumbens volume, in males. Development of the superior frontal cortex in males mediated the relationship between maternal aggressive behaviour and measures of late adolescent functioning. We suggest that our results support an association between negative parenting and adolescent functioning, which may be mediated by immature or delayed brain maturation.

## Introduction

Adverse childhood environments represent an important risk factor for the development of psychopathology later in life [1], and there is accumulating evidence from human and animal research that neurobiological changes may partially mediate this relationship [2, 3]. Indeed, there has been a recent surge of interest in the effects of adverse childhood environments on the brain, with a number of recent reviews highlighting the deleterious effects of adverse early environments on brain structure [4–6].

Although much research has focussed on relatively extreme forms of adverse environments, such as child abuse, less human work has investigated the effects of variation in parenting behaviors that might be considered less severe and more subtle. This is despite the fact that such variation in parenting behavior has long been suggested as critical for child development [7, 8]. Indeed, in a recent review, Belsky and de Haan [9] suggest that research on the influence of the environment on the brain has reached “the end of the beginning”, and that “work is now needed to determine whether and how variation in parenting in the normal range affects the brain development of children not exposed to extreme adversity” (p 423). Much animal literature supports associations between variations in maternal care and offspring brain development and behavior [see 10, 11]. For example, increased maternal licking/grooming behavior in rodents has been linked with decreased offspring anxiety and associated changes in function of the prefrontal cortex [12]. Maternal presence has been shown to alter rodent pup’s fear learning via social buffering blockade of amygdala plasticity [13]. In humans, we and others have found that negative, harsh or punitive parenting, and lower levels of positive, warm or supportive parenting, prospectively predict child emotional and behavioral problems that persist into adolescence and adulthood [14, 15]. Further, recent research provides evidence for links between *normative* variation in parenting behaviors/characteristics and child brain structure and function in regions including the prefrontal cortex [16–19], hippocampus [17, 20, 21], amygdala [19], and striatum [17].

While there is emerging evidence that parenting practices are associated with brain structure in humans, few studies have assessed measures of brain *development*. Such research is crucial for understanding how the neurobiological effects of parenting might unfold over time. This is particularly critical for the adolescent period, where brain maturation is dynamic, and measures of brain *change* may be particularly informative about neural insults. Indeed, our work has shown positive maternal behavior directed to the child to be associated with an acceleration of the normal pattern of cortical thinning in the prefrontal cortex during early adolescence [22]. Further, other research has shown that structural brain development during adolescence is related to cognitive and emotional functioning [23–25]. It is of note that in these studies, accelerated maturation post puberty, or an exaggeration of the normative pattern of growth (e.g., cortical thinning in the prefrontal cortex), generally appears to reflect positive development (i.e., is associated with superior functioning). For example, exaggerated thinning in the ventromedial prefrontal cortex has been associated with less depressive/anxiety symptoms in adolescents [23]. Exaggerated thinning of the anterior cingulate cortex during adolescence has been associated with greater inhibitory control [26]. Less research has investigated predictors and correlates of subcortical development, and the existing literature base does not point to a clear pattern of ‘adaptive’ development. For example, we previously found that positive parenting was associated with attenuated growth of the amygdala in early adolescence, while aggressive parenting was associated with exaggerated growth of the putamen [22].

The aim of the current study was to examine the effects of negative and positive parenting during early adolescence on structural brain development across the entire adolescent period using a multi-wave longitudinal design. Unlike cross-sectional studies, which are common in the brain development literature, longitudinal methods can make specific inferences about intraindividual change. Further, when assessing effects across large age spans, effects observed in longitudinal studies are more likely due to age as opposed to cohort effects [27]. We utilized observational measures of maternal positive behaviors during a conflict resolution task with their adolescent child, and maternal aversive/aggressive behaviors during a pleasant event planning task. These ‘out of context’ behaviors are likely to be particularly important indicators of mothers’ own emotionality (and emotion regulation), which have been suggested to shape adolescents’ emotion regulatory abilities and social competence [28]. These behaviors thus have implications for adolescent functioning in a range of life domains. For example, these specific maternal behaviors have been shown to predict child mental health outcomes, such as onset of depressive disorder [14]. As such, it is of particular interest to assess their impact on brain development.

This study builds on our earlier work in important ways; first, we examined brain development across the entire adolescent period, from age 11 to 20 (whereas our previous work has only examined development during the early to mid-adolescent period). Second, we investigated the effects of parenting behaviors on brain development using a whole-brain vertex-wise approach (rather than restrict analyses to a small number of regions of interest (ROIs). Third, we investigated how brain changes associated with parenting were in turn related to adolescent functioning. We hypothesized that a lower frequency of aggressive maternal behavior, and a higher frequency of positive maternal behavior, would predict an exaggeration of the normal pattern of growth of cortical thickness, particularly in prefrontal regions previously implicated in cross-sectional research on parenting and child brain structure. Due to the relative lack of prior research on environmental impacts on subcortical brain development, while we hypothesized that positive and negative parenting would be associated with the development of medial temporal and basal ganglia volumes, we did not make specific hypotheses about the direction of association. Finally, we hypothesized that the neurodevelopmental patterns associated with parenting will in turn be associated with behavioral functioning during late adolescence.

## Materials and Methods

### Participants and Recruitment

The sample described in the current study was derived from a larger (N = 2453) Australian longitudinal cohort. Based on their scores on the Early Adolescent Temperament Questionnaire-Revised (EATQ-R, [29]), 415 year six primary school students was selected to be part of the study, which has previously been described in detail by Yap and colleagues [30]. Adolescents at the extreme ends of the temperamental distribution were oversampled to maximize inter-individual differences in psychological well-being.

Of the selected adolescents, 245 agreed to participate in further research, and were invited to take part in brain Magnetic Resonance Imaging (MRI) assessments at three time points, when they were aged approximately 12, 16 and 19, respectively. Participants were assessed for Axis 1 disorders at each of these time points using the Schedule for Affective Disorder and Schizophrenia for School-Aged Children: Present and Lifetime Version. Socioeconomic classification (SES) was assessed based on the Australian National University Four (ANU_4_) Scale [31]. Maternal depressive symptoms were assessed at the first time point using the Centre for Epidemiological Studies Depression Scale (CES-D, [32]). A number of adolescents declined participation in the MRI assessments, resulting in 177 participants completing an MRI assessment at one or more time points. Based on visual inspection of processed MRI data by a researcher trained in neuroanatomy, nine of these participants were excluded due to poor MRI image quality and parcellation. In addition, two participants with full scale intelligence quotient (IQ) lower than 70 were excluded from analyses.

Following exclusions, 166 participants (n = 86 males) aged 11 to 20 years were available for analysis. Seventy-three of these participants had three scans, 55 had two scans and 38 had one scan. Males and females did not differ on the demographic or maternal behavior variables listed in Table 1 (all *p* values > 0.05). The final sample exhibited normal distribution on all temperamental factors (i.e. Negative Affectivity, Effortful Control, Surgency, Affiliation; *p* > 0.05), suggesting that the sampling bias used for recruiting participants had “re-normalized”. The final sample also did not differ from the initial school screening sample (N = 2453) on socioeconomic disadvantage (*t*_(2439)_ = -1.053; *p* = 0.29) or sex (Pearson’s *χ*^2^ = 1.963; *p* = 0.743). Twenty-eight participants of the final sample met the criteria for past or current psychiatric disorder at Time 1 (T1). An additional 28 participants met criteria for psychiatric diagnoses at Time 2 (T2), and 19 participants at Time 3 (T3). The prevalence of psychopathology in the sample is consistent with previous reports in large community samples [33]. We did not exclude participants with psychopathology because we wanted to model the effects of parenting on brain development in a normative community sample of adolescence, rather than an unrepresentative healthy sample. Refer to S1 Table for further detail on psychiatric diagnoses. However, we did investigate the influence of psychiatric diagnosis on results, described below. Written informed consent was obtained from the child and at least one parent/guardian at each time point. The research was approved by the Human Research Ethics Committee at The University of Melbourne, Australia.

**Table 1:** Sample characteristics.

	Sex		
	Male	Female	Total
T1 age (years)	12.83; 0.452	12.77; 0.394	12.79; 0.425
T2 age (years)	16.70; 0.559	16.71; 0.480	16.70; 0.518
T3 age (years)	19.10; 0.507	19.05; 0.413	19.08; 0.460
Delay time 1-2 (years)	3.80; 0.158	3.87; 0.237	3.83; 0.204
Delay time 2-3 (years)	2.40; 0.177	2.35; 0.251	2.38; 0.219
Estimate Full Scale IQ	107.96; 15.51	107.75; 15.80	107.86; 15.60
SES	58.14; 20.42	58.01; 21.36	58.08; 20.80
Maternal aggression RPM	0.55; 0.33	0.62; 0.39	0.58;0.36
Maternal positivity RPM	1.75; 0.60	1.68; 0.61	1.71; 0.62

NB: Values represent mean; standard deviation.

RPM = rate per minute, T1 = Time 1, T2 = Time 2, T3 = Time 3, IQ = intelligence quotient, SES = socioeconomic status.

### Family interaction assessment and measures

Adolescents and mothers completed the lab-based interaction assessment at T1. Mother-adolescent dyads completed two 20-min interaction tasks that were video recorded for subsequent coding. An event-planning interaction (EPI) was completed first, followed by a problem-solving interaction (PSI). The EPI and PSI tasks were intended to differentially elicit positive and negative behavior, respectively. The Living in Family Environments (LIFE) coding system [34] was used to code verbal and non-verbal maternal behavior from the video-recorded interactions. Codes were used to develop composite aggressive and positive behavior constructs, Measures of frequency (i.e., average number of times a mother expressed each behavior type per minute) of aggressive behavior during the EPI, and of positive behavior during the PSI were used in analyses. See S1 Methods and Results for more details about the coding procedure.

### Outcome measures

The child global assessment scale (CGAS, [35]) was administered to adolescent participants by trained research assistants to assess general functioning during late adolescence. The CGAS has no subscales but rather results in a total score from one to 100, with higher scores indicating superior functioning across a range of domains (i.e., functioning at school, home and with peers, involvement in activities and hobbies, absence of behavioral disturbance and psychiatric symptoms). During late adolescence, as part of the CGAS assessment, information was also collected pertaining to academic functioning (12^th^/senior year completion and Australian Tertiary Admission Rank (ATAR) scores [a percentile ranking of high school graduates’ final assessment performance], if applicable).

### MRI acquisition and analysis

#### Image acquisition

At T1, MRI scans were performed on a 3 Tesla GE scanner at the Brain Research Institute, Austin and Repatriation Medical Centre, Melbourne, Australia, with the following parameters: repetition time = 36 msec; echo time = 9msec; flip angle = 35°, field of view = 20cm, 124 T1-weighted contiguous slices (voxel dimensions = 0.4883 x 0.4883 x 1.5mm). MRI scans at T2 and T3 were performed on a 3 Tesla Siemens scanner at the Royal Children’s Hospital, Melbourne, Australia, with the following parameters: repetition time = 1900 msec; echo time = 2.24 msec; flip angle = 9°, field of view = 23cm; 176 T1-weighted contiguous slices (voxel dimensions = 0.9mm^3^). Although different scanners were used at T1 and T2, no inter-scanner bias was found (see S1 Methods and Results, S2 Table and S1 Fig for more details).

#### Image processing

Cortical reconstruction was performed using the longitudinal stream of FreeSurfer version 5.3 [36], which creates a within-unbiased subject template space and average image from both time points using robust, inverse consistent registration [37]. Cortical thickness values were automatically quantified within FreeSurfer on a vertex-by-vertex basis by computing the average shortest distance between the white matter boundary and the pial surface [38]. Subcortical volumes were estimated using an automated subcortical segmentation procedure that involves the assignment of a neuroanatomical label to each voxel in a MRI volume using a probabilistic atlas and Bayesian classification rule for label assignment. See S1 Methods and Results for further details about image processing.

### Statistical analysis

For cortical thickness, all statistical analyses were conducted in Matlab R2012a using the Freesurfer toolbox [39]. Separate linear mixed models were used to investigate the effect of maternal aggressive and positive behavior (during the EPI and PSI, respectively) on cortical development. Linear mixed modelling is an advanced technique for modelling within-person systematic change and between-person differences in development across different measurement waves over time. It is a flexible approach that can handle unbalanced repeated measurements with missing data. For each vertex of the cortical reconstruction, we fitted full analytic models to investigate the quadratic and linear effects of age, as well as interactions with sex and maternal behavior. In addition, SES was included as a covariate. Full models were represented by the following equation (mb = maternal behavior):

$$\begin{array}{l} Intercept+d_{ij}+\beta_{1}\left(age\right)+\beta_{2}\left(sex\right)+\beta_{3}\left(SES\right)+\beta_{4}\left(mb\right)+\beta_{5}\left(age^{2}\right)+\beta_{6}\left(age*sex\right)+\beta_{7}\left(age*mb\right)+\beta_{8}\left(sex*mb\right)+\\ \beta_{9}\left(age^{2}*sex\right)+\beta_{10}\left(age^{2}*mb\right)+\beta_{11}\left(age*sex*mb\right)+\beta_{12}\left(age^{2}*sex*mb\right)+e_{ijk} \end{array}$$

The d_ij_ term represents the random effect of the intercept within each vertex (j) in each subject (i). The e_ijk_ represents the normally distributed residual error term. Age, sex, SES, and maternal behavior effects were fixed, with β representing the parameter estimates for each of the main effects and interactions. All models were run with mean-centered age and maternal behavior terms. Full models were reduced using a top-down method based on the significance of higher-order terms. Significant effects (that survived False Discovery Rate correction of p < 0.05) were then visualized within FreeSurfer.

For subcortical volumes (left and right amygdala, hippocampus, caudate, putamen, pallidum, thalamus, nucleus accumbens), analyses were conducted using SPSS version 20 and results were considered significant at p < 0.0154 (corrected for multiple comparisons using false discovery rate p < 0.05) [40]. Similar linear mixed models (as described above for cortical thickness) were used to analyze the data, with separate models employed for each ROI.

Although we included participants with a history of psychopathology so that findings were representative of a community sample, we conducted follow-up analyses on significant results to investigate the possible influence of psychiatric diagnoses. Specifically, significant models were re-analyzed with the inclusion of lifetime disorder (present vs absent) as a covariate. Further, it might be argued that any effects of maternal behavior on adolescent brain development might be better explained by other factors that might influence parenting behavior, such as maternal mental health, or patterns of adolescent interactional behaviors that elicit specific parent behaviors. As such, the follow-up analyses also included maternal depressive symptoms and adolescent behavior during the interactions (i.e., frequency of adolescent aggressive behavior for analyses of maternal aggressive behavior, and frequency of adolescent positive behavior for analyses of maternal positive behavior) as covariates.

For any region whose development was found to be associated with maternal behaviour, mixed models were employed to assess whether development of this brain region was associated with late adolescent functional outcomes.

## Results

### Cortical thickness

**Fig 1:**
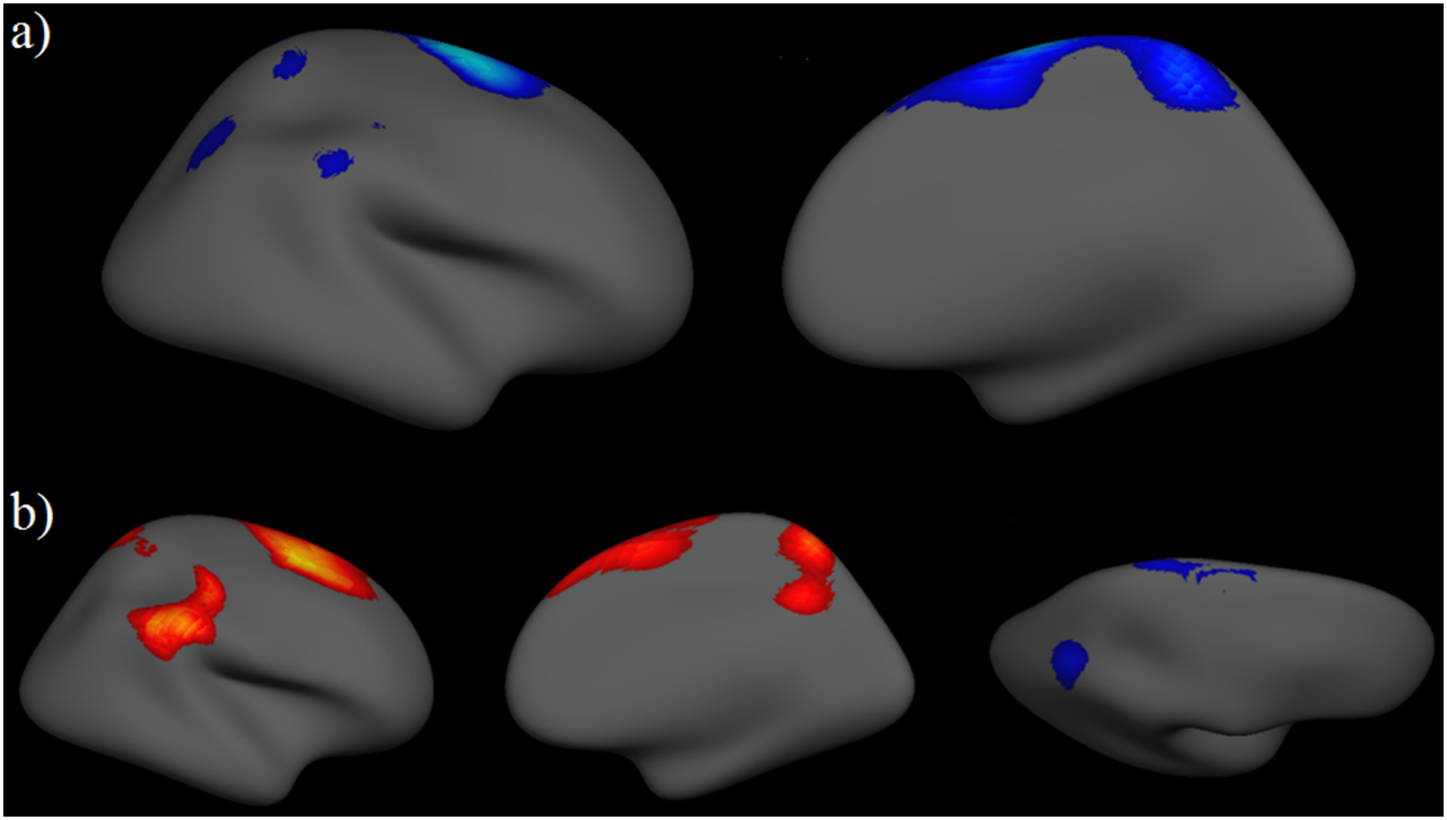
Association between mother aggressive behavior and brain development. a) Mother aggressive behavior by age by sex interactions for right hemisphere cortical thickness (FDR corrected, p < 0.05), and b) mother aggressive behavior by age interactions for male cortical thickness (left two images, FDR corrected, p < 0.05), and for female cortical thickness (right image, uncorrected, p < 0.001).

**Fig 2:**
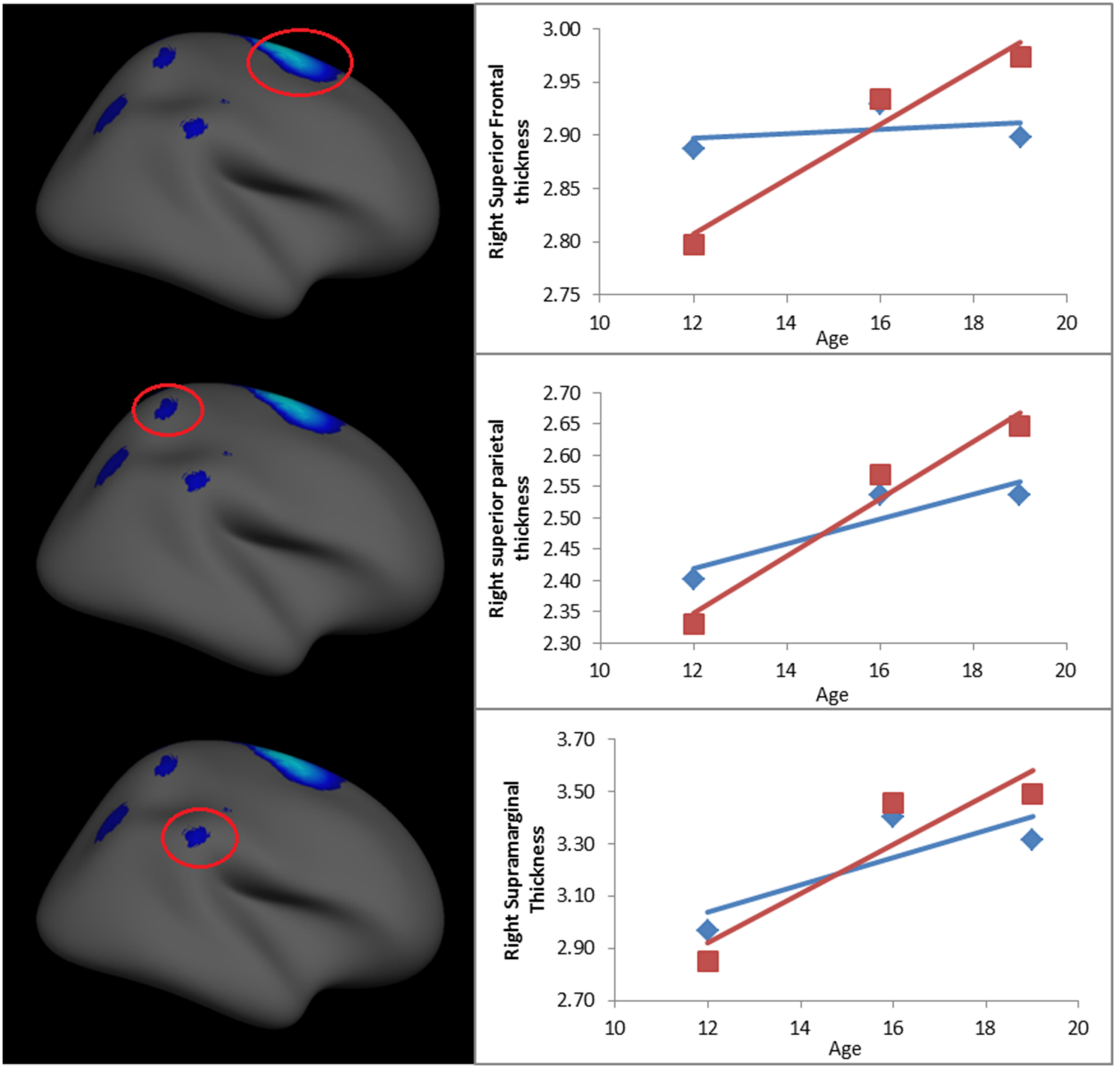
Mother aggressive behavior by age interaction for clusters that survived FDR (p < 0.05) correction for males. Data points represent predicted volume at each time point, and red and blue lines represent fitted age curves, for adolescents with high and low aggressive mothers, respectively.

No significant interactions between the quadratic effect of age, sex and maternal behavior were found. However, analyses revealed a significant 3-way interaction between age, sex and maternal aggressive behavior in the prediction of cortical thickness in the right hemisphere, indicating that maternal aggressive behavior predicted linear age-related changes in cortical brain development differently for males and females (see Fig 1a). Separate analyses by sex revealed that age by maternal aggressive behavior effects were present in males, but not females, in the superior frontal gyrus, supramarginal gyrus and superior parietal lobe (see Fig 1b). There were some age by maternal aggressive behavior effects evident in females (superior and inferior parietal lobe), but these did not survive correction for multiple comparisons (see Fig 1b). Average thickness from each significant cluster that survived correction for multiple comparisons was extracted and plotted in order to explore the nature of the interaction (see Fig 2 and Table 2). While analyses were performed on *continuous* maternal behavior measures, to facilitate ease of interpretation, high (+1 *SD*) and low (-1 *SD*) levels of maternal aggressive behavior were plotted.

**Table 2:** Results of significant models whereby maternal aggressive behavior predicted development of cortical thickness in males.

	Estimate	*t*	*p*
Right supramarginal			
age	0.05	5.45	< 0.001
Maternal aggression RPM	0.11	0.84	0.402
age x age	-0.02	-4.86	< 0.001
Maternal aggression RPM x age	0.06	2.64	0.011
Right superior frontal			
age	0.01	3.17	0.002
Maternal aggression RPM	0.01	0.10	0.918
age x age	-0.002	-2.13	0.036
Maternal aggression RPM x age	0.04	3.83	< 0.001
Right superior parietal			
age	0.03	4.97	< 0.001
Maternal aggression RPM	0.05	0.56	0.576
age x age	-0.004	-2.16	0.034
Maternal aggression RPM x age	0.04	2.50	0.014

NB: Models without covariates are not presented for simplicity.

RPM = rate per minute.

As can be seen from the data points in Fig 2 and statistics in Table 2, for males, cortical thickness in the superior frontal gyrus, superior parietal lobe and supramarginal gyrus developed in a quadratic pattern over adolescence, with thickening from early to mid-adolescence, and flattening or thinning from mid- to late adolescence (note that model fit statistics from mixed models without predictors/covariates confirmed that quadratic age models fit the data better than linear age models for the superior frontal gyrus and supramarginal gyrus ROIs, whereas a linear age model was the best fitting model for the superior parietal lobe ROI). Fig 2 illustrates that those males with mothers who displayed a higher frequency of aggressive behavior during early adolescence, had exaggerated increases in cortical thickness in the superior frontal gyrus, superior parietal lobe and supramarginal gyrus from early to late adolescence, compared to those males with mothers who displayed relatively less aggressive behavior.

Regarding maternal positive behavior, whole brain vertex-wise mixed model analyses showed that there were no main effects or interactions with age that predicted cortical thickness in either right or left hemisphere.

### Subcortical Volumes

**Fig 3:**
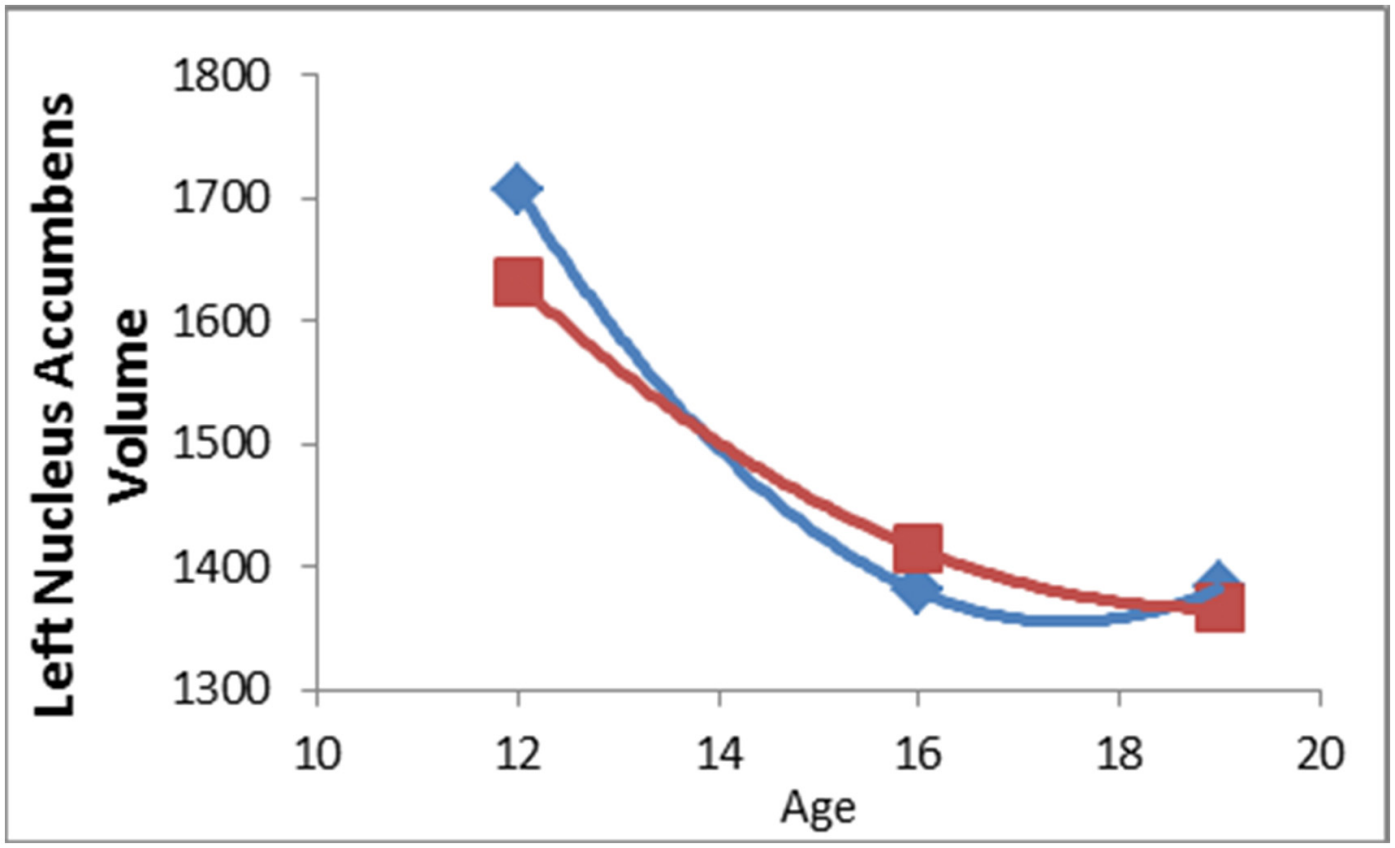
Mother aggressive behavior by quadratic age interaction for male left nucleus accumbens volume. Data points represent predicted volume at each time point, and red and blue lines represent fitted age curves, for adolescents with high and low aggressive mothers, respectively.

There was a significant interaction between the quadratic effect of age, sex and maternal aggressive behavior predicting left nucleus accumbens (NAcc) volume (β = 12.77, t = 2.56, p = 0.012). Separate analyses for each sex revealed a significant quadratic age by maternal aggressive behavior effect for males (β = -9.65, t = -2.60, p = 0.012) but not females (β = 3.21, t = 0.97, p = 0.337). As can be seen from the data points in Fig 3, for males, left NAcc volume developed in a quadratic pattern over adolescence, with marked decreases from early to mid-adolescence, and flattening or slowed decrease from mid- to late adolescence (note that model fit statistics from mixed models without predictors/covariates confirmed that a quadratic age model fit the data better than a linear age model for this region). Fig 3 illustrates that males whose mothers behaved relatively less aggressively showed a steep decrease in NAcc volume from early to mid-adolescence, followed by a deceleration in this pattern of decrease from mid- to late adolescence. On the other hand, males whose mothers behaved relatively more aggressively showed a flatter volume decrease from early to late adolescence. There were no other parenting main effects or interactions with age that predicted subcortical volume in either right or left hemisphere.

### Covariates

Controlling for maternal depressive symptoms, child aggressive/positive behavior, and psychiatric diagnosis did not change the pattern of significant and non-significant effects reported above.

### Functional outcomes

The association between brain development and adolescent outcomes was investigated for those brain regions whose development was significantly associated with parenting (i.e., male right superior frontal gyrus, superior parietal lobe and supramarginal gyrus, and male left nucleus accumbens). Mean thickness for cortical clusters was extracted for use in mixed model analyses. Mixed model analyses showed that linear developmental changes in the superior frontal region in males (i.e., changes identified to be associated with maternal aggressive behavior) were associated with final year school completion (t = 2.41, p = 0.017) and with global functioning (t = -2.09, p = 0.039). These results remained after controlling for SES, maternal aggressive behaviour, maternal depressive symptoms, child aggressive behavior and history of psychiatric diagnosis. Relative thickening of the superior frontal region over time was associated with poorer global functioning and lower rates of school completion. Of note, while maternal aggressive behavior during early adolescence was not directly associated with global functioning in late adolescence for males, relative superior frontal thickening partially mediated the relationship between higher aggressive maternal behavior and school non-completion (Sobel statistic = -1.96, p = 0.049), and there was a trend for relative superior frontal thickening to partially mediate the relationship between higher aggressive maternal behavior and poorer global functioning (Sobel statistic = -1.74, p = 0.082). For no other ROI was development associated with functional outcomes. See S1 Methods and Results for details about all associations between maternal behavior and late adolescent functioning variables.

## Discussion

In this multi-wave within-subjects longitudinal investigation of the association between parenting and adolescent brain development, we found that a higher frequency of observed aggressive maternal behaviors predicted greater thickening of the right superior frontal gyrus and areas of the right lateral parietal lobe in males. For the right superior frontal gyrus, this pattern was predictive of poor functional outcomes in late adolescence. Higher frequency of maternal aggressive behavior also predicted a reduced decline in left NAcc volume in males.

Consistent with hypotheses, maternal aggressive behavior was associated with development in the prefrontal cortex (specifically, the right superior frontal gyrus), however, it was also associated with development of thickness in non-hypothesized regions in the right lateral parietal cortex. This pattern of findings is consistent with previous research investigating the effects of some forms of childhood adversity on brain structure and function. For example, childhood adversity has been found to be negatively associated with cortical volume in a neural network that includes frontal and parietal regions [41]. Further, a recent functional study found parental criticism to be associated with decreased activity in the right temporoparietal junction extending to inferior parietal lobe [42].

The right superior frontal gyrus and lateral parietal cortex, including the supramarginal gyrus, are understood to be involved in executive functions, particularly related to attention and response inhibition [43]. The right supramarginal gyrus, part of the temporoparietal junction, is also thought to be an important area for social cognition [42]. These executive functions are understood to continue to mature during adolescence [44–46], and have been shown to be associated with adolescent brain development. For example, Tamnes and colleagues [47] found that superior response inhibition was associated with relatively greater thinning of the lateral parietal cortex during adolescence, and Luna and colleagues [48] found functional activation changes from childhood to adulthood during a response inhibition task in lateral parietal regions, including the supramarginal gyrus. It is thus plausible that the changes in cortical thickness associated with maternal aggressive behavior may be associated with behavioral changes in certain types of executive functions. Indeed, we found evidence that maternal aggressive behavior-related change in the superior frontal cortex was associated with poor functional outcomes in late adolescence; specifically, poor general functioning and increased school non-completion rates. Thus, our results support a mediation effect such that aggressive parenting might indirectly influence poor adolescent outcomes via effects on cortical brain development. The mechanisms of this effect require further investigation (e.g., whether the link between brain development and functional outcomes is driven by changes in executive function).

Maternal aggressive behavior was associated with relatively increased thickening across all cortical regions. In the full male sample, these regions evidenced thickening (see S1 Methods and Results for full details), which is somewhat unexpected and inconsistent with other reports of predominant thinning during this age period (e.g., [49]). The discrepancy may be in part due to the fact that our study included a greater number of within-subject scans than previous cross-sequential brain development studies (44% of individuals had 3 scans, and 33% had 2 scans) and so we may have more accurately been able to estimate intraindividual change, with less confounding cohort effects. In any case, it is difficult to say exactly how thickening associated with maternal aggressive behavior represents an alteration of the normative pattern of growth. It may be that it reflects an immature or delayed pattern of cortical development, where cortical thickness is yet to peak. Moreover, ‘flatter’ slopes associated with lower levels of maternal aggressive behavior may correspond to a more mature pattern of cortical development, where cortical thickness has peaked and has started its normative trajectory of thinning.

The mechanism by which aggressive maternal behavior might lead to a delayed or immature pattern of cortical development is unknown. Speculatively, one mechanism might involve the neurobiological consequences of stress associated with the experience of threatening maternal behavior. Alternatively, the neurodevelopmental alterations associated with maternal aggressive behavior might better reflect neural changes associated with deprivation or neglect. That is, high levels of maternal aggressive behavior might reflect a lack of appropriate parental emotion socialization, whereby children have a limited capacity throughout development to learn to express and regulate positive and negative emotions. It has been suggested that exposure to cognitive and social deprivation in children may shape the structure of association cortices involved in complex social and cognitive processing, as a result of abnormal pruning of synaptic connections, or abnormal dendritic branching [6].

The sex differences identified across the cortex are striking. Other studies that have investigated the effects of parenting on child/adolescent brain structure have either found few sex differences [22], or have not investigated such effects [20, 21]. Our male-specific findings for the cortex are at odds with a recent review concluding that females are more sensitive to environmental stressors in terms of psychological and biological consequences [50]. However, there is research supporting an association between parenting and behavioral problems in males, specifically [51]. Given evidence that deficits in frontal and parietal mediated executive functions are associated with behavioral problems, including antisocial behavior [52], it is possible that our findings reflect a specific socio-biological pathway to behavioral problems in males. Important to note is that maternal aggression was not significantly higher for males compared to females, and as such, our findings are not likely attributable to sex differences in maternal parenting.

Maternal aggressive behavior was also associated with volumetric change in the left NAcc for males. Specifically, maternal aggressive behavior was associated with a minimization of the normative pattern of nonlinear decrease, as predicted. The NAcc is thought to have a primary role in reward-related processing, but is also thought to be involved in multiple distinct aspects of cognitive and affective behavior, including operant and emotional learning, response inhibition and behavioral flexibility [53]. While there were no functional consequences of our result, given the context in which maternal aggressive behavior was measured (i.e., during a positive event planning task), it may be that maternal aggressive behavior during this task is somewhat analogous to the absence of expected positive/rewarding maternal behavior, or even the absence of anticipation of such behavior, and that this in turn impacted on the development of reward-related brain regions.

Interestingly, maternal positive behavior had no effects on adolescent brain development. Given that maternal positive behavior has been prospectively linked to adolescent outcomes in ours and other data (e.g., [14]), it is possible that these relationships are mediated by biological development not captured in this study (e.g., brain function, other indices of structural development such as white matter connectivity).

### Limitations

While the current study has a number of strengths, including the longitudinal design, and the use of observed (rather than self-reported) measures of maternal affective behaviors, the results need to be considered in the context of some limitations. First, we were unable to include fathers in analyses, due to the low number of participating fathers in the study. Fathers play a significant role in the socialization of emotion in their children, although this may differ to that of mothers [54]. Second, as MRI scans were acquired multisite, there is a possibility of scanner/sequence bias affecting volumetric and thickness estimates. However, post-acquisition procedures were adopted to minimize scanner effects on the acquired images. Further, data presented in S1 Methods and Results, S2 Table and S1 Fig showed no interscanner bias for the ROIs in this study. While the actual trajectories of cortical thickness should be interpreted with caution, importantly, since all adolescents were assessed in the same scanner at each wave, different scanners would not influence longitudinal between-individual comparisons. That is, individual differences in maternal behavior could not have interacted with scanner type in any way that might bias the reported results.

### Conclusion

This is the first study to our knowledge to investigate the effects of parenting behaviors on adolescent brain development using a within-subjects multi-wave longitudinal design. We found that maternal aggressive behavior observed in early adolescence was associated with similar trajectories of development in the right superior frontal and lateral parietal cortices in males from early to late adolescence. The changes observed in the superior frontal cortex were in turn associated with poor functional outcomes in late adolescence. Maternal aggressive behavior also predicted development of the NAcc in males. We interpret these findings as potentially reflecting a negative effect of maternal aggression on adolescent brain development. Specifically, maternal aggression appears to be associated with delayed or immature maturation of specific brain regions that are thought to underlie aspects of cognitive and emotional functioning, and some of these changes might mediate a link between aggressive maternal behavior and poor outcomes in late adolescence and beyond. Further research is required to investigate whether the parenting-related brain changes observed in this study have implications for functioning, including the development of psychopathology, into adulthood.

## Supporting Information

S1 Fig Inter-scanner reliability analysis. Proportion of male and female participants for whom ROI thickness increased (green), decreased (blue) or did not change (red) based on the inter-scanner reliability analysis.
(DOCX)

S1 Methods and Results S1 Methods and Results provides more detailed information about methods (family interaction coding and MRI processing, including analysis of inter-scanner effects) and results (more comprehensive information about associations between maternal behaviors and adolescent outcomes). (DOC)

S1 Table Psychopathology characteristics of the sample. (DOCX)

S2 Table Average SD (mm) for each ROI based on individuals scanned at both sites for reliability analysis. (DOCX)

## References

[1] Heim C, Nemeroff CB. The role of childhood trauma in the neurobiology of mood and anxiety disorders: preclinical and clinical studies. Biological Psychiatry. 2001;49(12):1023–39. 10.1016/S0006-3223(01)01157-X. 11430844

[2] Tupler LA, De Bellis MD. Segmented hippocampal volume in children and adolescents with posttraumatic stress disorder. Biological psychiatry. 2006;59(6):523–9. 16199014 10.1016/j.biopsych.2005.08.007

[3] Howell BR, Grand AP, McCormack KM, Shi Y, LaPrarie JL, Maestripieri D, et al Early adverse experience increases emotional reactivity in juvenile rhesus macaques: Relation to amygdala volume. Developmental psychobiology. 2014;56(8):1735–46. 10.1002/dev.21237 25196846 PMC4433484

[4] Lupien SJ, McEwen BS, Gunnar MR, Heim C. Effects of stress throughout the lifespan on the brain, behaviour and cognition. Nature Reviews Neuroscience. 2009;10(6):434–45. 10.1038/nrn2639 19401723

[5] Hart H, Rubia K. Neuroimaging of child abuse: a critical review. Frontiers in human neuroscience. 2012;6. 10.3389/fnhum.2012.00052 PMC3307045 22457645

[6] McLaughlin KA, Sheridan MA, Lambert HK. Childhood adversity and neural development: deprivation and threat as distinct dimensions of early experience. Neuroscience & Biobehavioral Reviews. 2014;47:578–91. 25454359 10.1016/j.neubiorev.2014.10.012 PMC4308474

[7] Spera C. A review of the relationship among parenting practices, parenting styles, and adolescent school achievement. Educational Psychology Review. 2005;17(2):125–46.

[8] Bronfenbrenner U. Ecology of the family as a context for human development: Research perspectives. Developmental psychology. 1986;22(6):723.

[9] Belsky J, de Haan M. Annual Research Review: Parenting and children’s brain development: the end of the beginning. Journal of Child Psychology and Psychiatry. 2011;52(4):409–28. 10.1111/j.1469-7610.2010.02281.x 20626527

[10] Kaufman J, Plotsky PM, Nemeroff CB, Charney DS. Effects of early adverse experiences on brain structure and function: clinical implications. Biological psychiatry. 2000;48(8):778–90. 11063974 10.1016/s0006-3223(00)00998-7

[11] Francis DD, Meaney MJ. Maternal care and the development of stress responses. Current opinion in neurobiology. 1999;9(1):128–34. 10072372 10.1016/s0959-4388(99)80016-6

[12] Van Hasselt FN, De Visser L, Tieskens JM, Cornelisse S, Baars AM, Lavrijsen M, et al Individual variations in maternal care early in life correlate with later life decision-making and c-fos expression in prefrontal subregions of rats. PLoS One. 2012;7(5):e37820 10.1371/journal.pone.0037820 22693577 PMC3365050

[13] Moriceau S, Sullivan RM. Maternal presence serves as a switch between learning fear and attraction in infancy. Nature neuroscience. 2006;9(8):1004–6. 16829957 10.1038/nn1733 PMC1560090

[14] Schwartz OS, Byrne ML, Simmons JG, Whittle S, Dudgeon P, Yap MB, et al Parenting During Early Adolescence and Adolescent-Onset Major Depression A 6-Year Prospective Longitudinal Study. Clinical Psychological Science. 2014;2(3):272–86.

[15] Shaw DS, Winslow EB. Precursors and correlates of antisocial behavior from infancy to preschool. 1997.

[16] Frye RE, Malmberg B, Swank P, Smith K, Landry S. Preterm birth and maternal responsiveness during childhood are associated with brain morphology in adolescence. Journal of the International Neuropsychological Society. 2010;16(05):784–94. 20609271 10.1017/S1355617710000585

[17] Schneider S, Brassen S, Bromberg U, Banaschewski T, Conrod P, Flor H, et al Maternal interpersonal affiliation is associated with adolescents’ brain structure and reward processing. Translational psychiatry. 2012;2(11):e182. 23149446 10.1038/tp.2012.113 PMC3565762

[18] Tomoda A, Suzuki H, Rabi K, Sheu Y-S, Polcari A, Teicher MH. Reduced prefrontal cortical gray matter volume in young adults exposed to harsh corporal punishment. Neuroimage. 2009;47:T66–T71. 10.1016/j.neuroimage.2009.03.005 19285558 PMC2896871

[19] Whittle S, Yap MB, Yücel M, Sheeber L, Simmons JG, Pantelis C, et al Maternal responses to adolescent positive affect are associated with adolescents’ reward neuroanatomy. Social cognitive and affective neuroscience. 2009:nsp012. 10.1093/scan/nsp012 PMC2728631 19398536

[20] Rao H, Betancourt L, Giannetta JM, Brodsky NL, Korczykowski M, Avants BB, et al Early parental care is important for hippocampal maturation: evidence from brain morphology in humans. Neuroimage. 2010;49(1):1144–50. 10.1016/j.neuroimage.2009.07.003 19595774 PMC2764790

[21] Luby JL, Barch DM, Belden A, Gaffrey MS, Tillman R, Babb C, et al Maternal support in early childhood predicts larger hippocampal volumes at school age. Proceedings of the National Academy of Sciences. 2012;109(8):2854–9. 10.1073/pnas.1118003109 PMC3286943 22308421

[22] Whittle S, Simmons JG, Dennison M, Vijayakumar N, Schwartz O, Yap MB, et al Positive parenting predicts the development of adolescent brain structure: A longitudinal study. Developmental cognitive neuroscience. 2014;8:7–17. 10.1016/j.dcn.2013.10.006 24269113 PMC6990097

[23] Ducharme S, Albaugh MD, Hudziak JJ, Botteron KN, Nguyen T-V, Truong C, et al Anxious/depressed symptoms are linked to right ventromedial prefrontal cortical thickness maturation in healthy children and young adults. Cerebral Cortex. 2013:bht151. 10.1093/cercor/bht151 PMC4193463 23749874

[24] Shaw P, Greenstein D, Lerch J, Clasen L, Lenroot R, Gogtay N, et al Intellectual ability and cortical development in children and adolescents. Nature. 2006;440(7084):676–9. 16572172 10.1038/nature04513

[25] Vijayakumar N, Whittle S, Dennison M, Yücel M, Simmons J, Allen NB. Development of temperamental effortful control mediates the relationship between maturation of the prefrontal cortex and psychopathology during adolescence: A 4-year longitudinal study. Developmental cognitive neuroscience. 2014;9:30–43. 10.1016/j.dcn.2013.12.002 24486655 PMC6989743

[26] Vijayakumar N, Whittle S, Yücel M, Dennison M, Simmons J, Allen NB. Prefrontal Structural Correlates of Cognitive Control during Adolescent Development: A 4-Year Longitudinal Study. 2013. 10.1162/jocn_a_00549 24345180

[27] Schaie KW. 3 Developmental Designs Revisited. 1994.

[28] Yap MB, Allen NB, Sheeber L. Using an emotion regulation framework to understand the role of temperament and family processes in risk for adolescent depressive disorders. Clinical child and family psychology review. 2007;10(2):180–96. 17265137 10.1007/s10567-006-0014-0

[29] Capaldi DM, Rothbart MK. Development and validation of an early adolescent temperament measure. The Journal of Early Adolescence. 1992;12(2):153–73.

[30] Yap MB, Allen NB, O'Shea M, Di Parsia P, Simmons JG, Sheeber L. Early adolescents' temperament, emotion regulation during mother—child interactions, and depressive symptomatology. Development and psychopathology. 2011;23(01):267–82. 21262053 10.1017/S0954579410000787

[31] Jones FL, McMillan J. Scoring occupational categories for social research: A review of current practice, with Australian examples. Work, Employment & Society. 2001;15(3):539–63.

[32] Radloff LS. The CES-D scale a self-report depression scale for research in the general population. Applied psychological measurement. 1977;1(3):385–401.

[33] Merikangas KR, He J-P, Burstein M, Swanson SA, Avenevoli S, Cui L, et al Lifetime prevalence of mental disorders in US adolescents: results from the National Comorbidity Survey Replication—Adolescent Supplement (NCS-A). Journal of the American Academy of Child & Adolescent Psychiatry. 2010;49(10):980–9. 20855043 10.1016/j.jaac.2010.05.017 PMC2946114

[34] Hops H, Biglan A, Tolman A, Arthur J, Longoria N. Living in Family Environments (LIFE) coding system: Manual for coders (Revised). Eugene, OR: Oregon Research Institute. 1995.

[35] Shaffer D, Gould MS, Brasic J, Ambrosini P, Fisher P, Bird H, et al A children's global assessment scale (CGAS). Archives of General psychiatry. 1983;40(11):1228–31. 6639293 10.1001/archpsyc.1983.01790100074010

[36] Reuter M, Schmansky NJ, Rosas HD, Fischl B. Within-subject template estimation for unbiased longitudinal image analysis. Neuroimage. 2012;61(4):1402–18. 10.1016/j.neuroimage.2012.02.084 22430496 PMC3389460

[37] Reuter M, Fischl B. Avoiding asymmetry-induced bias in longitudinal image processing. Neuroimage. 2011;57(1):19–21. 10.1016/j.neuroimage.2011.02.076 21376812 PMC3260043

[38] Fischl B, Dale AM. Measuring the thickness of the human cerebral cortex from magnetic resonance images. Proceedings of the National Academy of Sciences. 2000;97(20):11050–5. 10.1073/pnas.200033797 PMC27146 10984517

[39] Bernal-Rusiel JL, Greve DN, Reuter M, Fischl B, Sabuncu MR. Statistical analysis of longitudinal neuroimage data with linear mixed effects models. Neuroimage. 2013;66:249–60. 10.1016/j.neuroimage.2012.10.065 23123680 PMC3586747

[40] Benjamini Y, Yekutieli D. The control of the false discovery rate in multiple testing under dependency. The Annals of Statistics. 2001;29(4):1165–88.

[41] Walsh ND, Dalgleish T, Lombardo MV, Dunn VJ, Van Harmelen A-L, Ban M, et al General and specific effects of early-life psychosocial adversities on adolescent grey matter volume. NeuroImage: Clinical. 2014;4:308–18. 25061568 10.1016/j.nicl.2014.01.001 PMC4107373

[42] Lee KH, Siegle GJ, Dahl RE, Hooley JM, Silk JS. Neural responses to maternal criticism in healthy youth. Social cognitive and affective neuroscience. 2014:nsu133. 10.1093/scan/nsu133 PMC4483556 25338632

[43] Fan J, McCandliss BD, Fossella J, Flombaum JI, Posner MI. The activation of attentional networks. Neuroimage. 2005;26(2):471–9. 15907304 10.1016/j.neuroimage.2005.02.004

[44] Luna B, Garver KE, Urban TA, Lazar NA, Sweeney JA. Maturation of cognitive processes from late childhood to adulthood. Child development. 2004;75(5):1357–72. 15369519 10.1111/j.1467-8624.2004.00745.x

[45] Anderson VA, Anderson P, Northam E, Jacobs R, Catroppa C. Development of executive functions through late childhood and adolescence in an Australian sample. Developmental neuropsychology. 2001;20(1):385–406. 11827095 10.1207/S15326942DN2001_5

[46] Choudhury S, Blakemore S-J, Charman T. Social cognitive development during adolescence. Social cognitive and affective neuroscience. 2006;1(3):165–74. 10.1093/scan/nsl024 18985103 PMC2555426

[47] Tamnes CK, Østby Y, Walhovd KB, Westlye LT, Due-Tønnessen P, Fjell AM. Neuroanatomical correlates of executive functions in children and adolescents: A magnetic resonance imaging (MRI) study of cortical thickness. Neuropsychologia. 2010;48(9):2496–508. 10.1016/j.neuropsychologia.2010.04.024. 20434470

[48] Luna B, Thulborn KR, Munoz DP, Merriam EP, Garver KE, Minshew NJ, et al Maturation of widely distributed brain function subserves cognitive development. Neuroimage. 2001;13(5):786–93. 11304075 10.1006/nimg.2000.0743

[49] Shaw P, Kabani NJ, Lerch JP, Eckstrand K, Lenroot R, Gogtay N, et al Neurodevelopmental trajectories of the human cerebral cortex. The Journal of Neuroscience. 2008;28(14):3586–94. 10.1523/JNEUROSCI.5309-07.2008 18385317 PMC6671079

[50] Oldehinkel AJ, Bouma E. Sensitivity to the depressogenic effect of stress and HPA-axis reactivity in adolescence: a review of gender differences. Neuroscience & Biobehavioral Reviews. 2011;35(8):1757–70. 21040743 10.1016/j.neubiorev.2010.10.013

[51] Rothbaum F, Weisz JR. Parental caregiving and child externalizing behavior in nonclinical samples: a meta-analysis. Psychological bulletin. 1994;116(1):55 8078975 10.1037/0033-2909.116.1.55

[52] Cauffman E, Steinberg L, Piquero AR. Psychological, Neuropsychological and Physiological Correlates of Serious Antisocial Behavior in Adolescence: The Role Of Self-Control*. Criminology. 2005;43(1):133–76.

[53] Goto Y, Grace AA. Limbic and cortical information processing in the nucleus accumbens. Trends in neurosciences. 2008;31(11):552–8. 10.1016/j.tins.2008.08.002 18786735 PMC2884964

[54] Sheeber LB, Davis B, Leve C, Hops H, Tildesley E. Adolescents' relationships with their mothers and fathers: associations with depressive disorder and subdiagnostic symptomatology. Journal of abnormal psychology. 2007;116(1):144 17324025 10.1037/0021-843X.116.1.144 PMC2249923

