# Supplementary material for: Observed Measures of Negative Parenting Predict Brain Development during Adolescence
Source: PLoS One. 2016 Jan 29;11(1):e0147774. doi: 10.1371/journal.pone.0147774 (PMC4732618; doi:10.1371/journal.pone.0147774)
Supplement: S1 Fig — Proportion of male and female participants for whom ROI thickness increased (green), decreased (blue) or did not change (red) based on the inter-scanner reliability analysis. (DOCX) [file pone.0147774.s001.docx]

**S1 Fig. Proportion of male and female participants for whom ROI thickness increased (green), decreased (blue) or did not change (red) based on the inter-scanner reliability analysis.** Proportions are consistent with statistical tests of change in thickness in each region for males and females. Superior parietal: Significant increase in males (p < 0.001); no change in females (p = 0.313); Inferior parietal: Significant decrease in both males (p = 0.036) and females (0.001); Supramarginal: Significant increase in both males and females (p’s < 0.001); Superior frontal: Significant increase in males (p < 0.001); no change in females (p = 0.916).
