# Supplementary material for: Observed Measures of Negative Parenting Predict Brain Development during Adolescence
Source: PLoS One. 2016 Jan 29;11(1):e0147774. doi: 10.1371/journal.pone.0147774 (PMC4732618; doi:10.1371/journal.pone.0147774)
Supplement: S1 Methods and Results — (DOC) [file pone.0147774.s002.doc]

**S1 Methods/Results**

Methods

*Family interaction assessment and measures*

Adolescents and mothers completed the lab-based interaction assessment at T1. Mother-adolescent dyads completed two 20-min interaction tasks that were video recorded for subsequent coding. An event-planning interaction (EPI) was completed first, followed by a problem-solving interaction (PSI). The EPI and PSI tasks were intended to differentially elicit positive and negative behavior, respectively. For the EPI, mothers and adolescents were instructed to plan one or more pleasant activities to do together, with up to five activities chosen on the basis of items that both the mother and adolescent rated as being “very pleasant” on the Pleasant Events Schedule . For the PSI, mother-adolescent dyads were instructed to try to resolve one or more issues of disagreement, with up to five issues selected that the mother and adolescent endorsed as occurring the most frequently and generating the highest intensity of anger on the Issues Checklist .

*Living in Family Environments (LIFE) coding system*

The LIFE is an observational, microsocial coding system that allows for a detailed analysis of individual family members’ behaviors. The LIFE system consists of 10 nonverbal affect codes (e.g., anger, dysphoria, happy) and 27 verbal content codes (e.g., validation, complaint, provoke). To code the video-recorded interactions, we used an event-based protocol in which new codes were entered each time the affect or content of one of the interactants changed. The affect and content codes were used to develop composite behavior constructs. In this study, the constructs were aggressive and positive behaviors. The aggressive construct included all behaviors with contemptuous, angry, or belligerent affect, as well as cruel, provocative, annoying/disruptive, or argumentative verbal statements made with neutral affect. The positive construct included all behaviors with happy or caring affect as well as approving, validating, affectionate, or humorous comments made with neutral affect. We used the LIFE data to construct a frequency variable to measure maternal expression of emotion. These variables indicate the average number of times a mother expressed each behavior type per minute and were calculated separately for the EPI and PSI. Frequency of aggressive behavior during the EPI, and of positive behavior during the PSI were used in analyses. Coders were extensively trained and blind to the clinical and demographic characteristics of the participants. Approximately 20% of the interactions were coded by a second observer to provide an estimate of observer agreement. Kappa reliability coefficients for the Aggressive and Positive constructs were 0.70 and 0.86, respectively.

*MRI acquisition and analysis*

*Image Acquisition.* At T1, MRI scans were performed on a 3 Tesla GE scanner at the Brain Research Institute, Austin and Repatriation Medical Centre, Melbourne, Australia, with the following parameters: repetition time = 36 msec; echo time = 9msec; flip angle = 35°, field of view = 20cm, 124 T1-weighted contiguous slices (voxel dimensions = 0.4883 x 0.4883 x 1.5mm). MRI scans at T2 and T3 were performed on a 3 Tesla Siemens scanner at the Royal Children’s Hospital, Melbourne, Australia, with the following parameters: repetition time = 1900 msec; echo time = 2.24 msec; flip angle = 9°, field of view = 23cm; 176 T1-weighted contiguous slices (voxel dimensions = 0.9mm^3^).

*Image Processing.* Images were transferred to an SGI/Linux workstation for morphometric analysis. Cortical reconstruction was performed using the FreeSurfer image analysis suite (http://surfer.nmr.mgh.harvard.edu/). Cortical thickness values were automatically quantified within FreeSurfer on a vertex-by-vertex basis by computing the average shortest distance between the white matter boundary and the pial surface . Surface boundaries were visually inspected by a trained rater and, if necessary, errors due to segmentation miss-classification were manually corrected and re-processed. Subcortical volumes were estimated using an automated subcortical segmentation procedure that involves the assignment of a neuroanatomical label to each voxel in a MRI volume using a probabilistic atlas and Bayesian classification rule for label assignment. Subcortical segmentation output was visually inspected for accuracy by an individual trained in neuroanatomy. In order to address issues arising from longitudinal and/or multisite studies (such as geometric distortion and voxel dimension drift), images were processed through the longitudinal stream of FreeSurfer version 5.3 , which creates a within-unbiased subject template space and average image from both time points using robust, inverse consistent registration . The template is used as an estimate to initialize subsequent segmentation processes in the longitudinal stream for each time point, providing common information regarding anatomical structures. This process significantly improves the repeatability and power of cortical measurements, having superior robustness with respect to noise, intensity scaling and outliers when compared to alternate registration tools . All FreeSurfer image processing was conducted on a high performance computing facility at the Melbourne Neuropsychiatry Centre, Melbourne, Australia.

*Inter-scanner reliability study*

Four individuals, aged 23, 28, 35 and 36 years were each scanned at RCH and BRI within a two-week period. The same acquisition parameters were used at each location to those described in the methods section, as well as the same semi-automated methods of processing to extract cortical thickness and subcortical volume. A reliability analysis was conducted on the regions where developmental change was associated with maternal behavior. The reliability analysis was based on a descriptive procedure proposed by Lebel and Beaulieu . They produced established thresholds for determining whether the amount of change observed in the study sample was likely to have occurred over and above that expected from scanner effects. Standard deviations for each ROI were calculated for each person within the reliability study (n = 4) based on their scores from each scanner. The group average standard deviation was then calculated for each ROI (mean SD across all subjects), which are listed in Table S2. These values provide estimates of the measurement variability in each ROI that can be expected from scanner differences alone. The average SD data was applied to the study sample in order to determine the proportion (i.e., percentage) of subjects that experienced greater change, either increases or decreases, than the average SD. For each subject, change for each ROI was calculated using a difference score (i.e., cortical thickness for time 2 – time 1). Those with difference scores within 1 SD (determined from the reliability study) were considered to not change, while those with difference scores greater than 1 SD were considered to experience true change (over and above scanner effects). When the majority of subjects experienced longitudinal change in the direction identified by the mixed models in the study sample, results were considered reliable and not due to scanner bias. The results from our sample, presented in figure S1, indicate that for each ROI the majority of individuals experienced change in thickness over time consistent with statistical tests of change. Thus, although different scanners were used at Time 1 and 2, we have shown no inter-scanner bias. Further, it is unlikely that the measures of maternal behavior would interact with scanner or sequence type in any way that might bias the results.

Results

*Associations between maternal behaviour variables and adolescent functional outcomes*

Regression analyses were performed to assess the association between parenting variables and behavioral outcomes. Analyses showed that after controlling for SES, maternal aggressive behavior during early adolescence was associated with lower global functioning in late adolescence (β = -0.35, t = -3.5, p = 0.001). Adolescent sex did not moderate this association (p = 0.265). Controlling for psychiatric diaognosis, adolescent aggressive behavior and maternal depressive symptoms did not alter the significant association between maternal aggressive behavior and global functioning (β = -0.29, t = -2.90, p = 0.005). After controlling for SES, maternal positive behavior during early adolescence was associated with higher global functioning in late adolescence (β = 0.24, t = 2.27, p = 0.025), and sex did not moderate the association (p = 0.432). However, after controlling for psychiatric diagnosis, adolescent aggressive behavior and maternal depressive symptoms, maternal positive behavior was no longer significantly associated with global functioning (β = 0.17, t = 1.88, p = 0.064). Neither maternal positive behavior nor maternal aggressive behavior was associated with late adolescent academic function (ATAR scores and final year completion), before or after including covariates of no interest (all p values > 0.14). Further, adolescent sex did not moderate any of these associations (all p values > 0.09).

References

1. MacPhillamy DJ, Lewinsohn PM. Manual for the pleasant events schedule: DJ MacPhillamy & PM Lewinsohn; 1976.

2. Prinz RJ, Foster S, Kent RN, O'Leary KD. Multivariate assessment of conflict in distressed and nondistressed mother‐adolescent dyads. J Appl Behav Anal. 1979;12(4):691-700.

3. Hops H, Biglan A, Tolman A, Arthur J, Longoria N. Living in Family Environments (LIFE) coding system: Manual for coders (Revised). Eugene, OR: Oregon Research Institute. 1995.

4. Fischl B, Dale AM. Measuring the thickness of the human cerebral cortex from magnetic resonance images. Proceedings of the National Academy of Sciences. 2000;97(20):11050-5.

5. Reuter M, Schmansky NJ, Rosas HD, Fischl B. Within-subject template estimation for unbiased longitudinal image analysis. Neuroimage. 2012;61(4):1402-18.

6. Reuter M, Fischl B. Avoiding asymmetry-induced bias in longitudinal image processing. Neuroimage. 2011;57(1):19-21.

7. Reuter M, Rosas HD, Fischl B. Highly accurate inverse consistent registration: a robust approach. Neuroimage. 2010;53(4):1181-96.

8. Lebel C, Beaulieu C. Longitudinal development of human brain wiring continues from childhood into adulthood. The Journal of Neuroscience. 2011;31(30):10937-47.
