# Supplementary material for: Observed Measures of Negative Parenting Predict Brain Development during Adolescence
Source: PLoS One. 2016 Jan 29;11(1):e0147774. doi: 10.1371/journal.pone.0147774 (PMC4732618; doi:10.1371/journal.pone.0147774)
Supplement: S1 Table — (DOCX) [file pone.0147774.s003.docx]

**S1 Table. Psychopathology characteristics of the sample**

|  | T1 | T1 - T2 | T2 - T3 |
| --- | --- | --- | --- |
| Depression | 3 | 18 | 19 |
| Anxiety | 16 | 11 | 12 |
| Attention | 6 | 0 | 0 |
| Behaviour | 6 | 10 | 1 |
| Substance | 0 | 9 | 14 |
| Eating | 0 | 1 | 1 |
| Adjustment | 0 | 4 | 5 |

NB: Values represent frequency

T1 = Time 1, T2 = Time 2, T3 = Time 3
