# Supplementary material for: Observed Measures of Negative Parenting Predict Brain Development during Adolescence
Source: PLoS One. 2016 Jan 29;11(1):e0147774. doi: 10.1371/journal.pone.0147774 (PMC4732618; doi:10.1371/journal.pone.0147774)
Supplement: S2 Table — (DOCX) [file pone.0147774.s004.docx]

**S2 Table. Average SD (mm) for each ROI based on individuals scanned at both sites for reliability analysis**

| *Structure* | *Average SD (mm)* |
| --- | --- |
| Right superior frontal | 0.086 |
| Right superior parietal | 0.124 |
| Right inferior parietal | 0.040 |
| Right supramarginal | 0.043 |

Note: Interscanner reliability information for the nucleus accumbens can be found in Dennison et al. ([2013](#_ENREF_1)). No bias was found and volumes were found to decrease for both male and females, consistent with results of mixed model analyses.
